# Monkeypox Virus Surveillance in Wastewater, North Carolina, USA, October 2023–May 2025

**DOI:** 10.3201/eid3213.260414

**Published:** 2026-08

**Authors:** Nicole L. Snyder, Thomas Clerkin, Sarah H. Koenigsberg, Mara B. Tate, Ty L. Lautenschlager, Justin Albertson, Emma L. Doran, Erica F. Wilson, Rachel T. Noble, Virginia T. Guidry, Ariel R. Christensen

**Affiliations:** Division of Public Health, North Carolina Department of Health and Human Services, Raleigh, North Carolina, USA (N.L. Snyder, S.H. Koenigsberg, M.B. Tate, T.L. Lautenschlager, J. Albertson, E.L. Doran, E.F. Wilson, V.T. Guidry, A.R. Christensen); Institute of Marine Sciences, University of North Carolina at Chapel Hill, Morehead City, North Carolina, USA (T. Clerkin, R.T. Noble); Gillings School of Global Public Health, University of North Carolina at Chapel Hill, Chapel Hill, North Carolina, USA (R.T. Noble)

**Keywords:** viruses, sexually transmitted infections, monitoring, mpox, MPXV, sensitivity, specificity, surveillance, wastewater, North Carolina

## Abstract

In September 2023, the North Carolina Department of Health and Human Services identified a new case of mpox clade II 5 months after the last previous report. To assess possible unrecognized transmission, nonvariola orthopoxvirus testing was implemented at select wastewater sites. During October 29, 2023–May 2025, a total of 801 samples were collected across 16 treatment plants serving ≈2 million residents. We analyzed wastewater detections (n = 47) and clinical cases (n = 45) using a +17-day window. Overall sensitivity of identifying the presence of >1 temporally aligned mpox case within a sewershed was 10.6% (95% CI 6.5%–16.7%); specificity was 95.1% (95% CI 93.2%–96.5%), positive predictive value was 31.9% (95% CI 20.4%–46.2%), and negative predictive value was 83.2% (95% CI 80.3%–85.7%). Restricting detections above the limit of detection reduced sensitivity but increased specificity. Overall, wastewater surveillance showed low sensitivity but provided complementary awareness for public health response.

Since cases were first identified outside mpox-endemic regions in 2022, mpox has emerged as an increasing public health concern in the United States. The global outbreak in 2022 affected 75 countries and territories; >2,300 cases had been confirmed in the United States by July 2022 (*1*). Because of evolving transmission dynamics, severity, and uncertainty surrounding social and economic impacts, the World Health Organization declared mpox a public health emergency of international concern on July 23, 2022 (*2*–*3*).

Mpox is an infectious disease endemic to regions of Central and West Africa. It is caused by the enveloped DNA virus monkeypox virus (MPXV) and is characterized by fever, headache, myalgia, lymphadenopathy, and a vesiculopustular rash that can involve the face, extremities, and genital region (*3*). Mpox is primarily transmitted through direct contact with active lesions, contaminated materials, bodily fluids, or respiratory secretions during close contact (*4*,*5*). Mpox infections are categorized into 2 genetically distinct clades, clade I and clade II. Clade II infections, which were responsible for the global outbreak that began in 2022, are generally associated with less severe disease and reduced transmissibility (*5*). Historically, clade I mpox infections have demonstrated greater virulence, mortality, and transmission (*6*).

The Centers for Disease Control and Prevention (CDC) National Wastewater Surveillance System initiated wastewater testing for MPXV in October 2022, five months after the mpox outbreak began in the United States. For underreported and stigmatized infections such as mpox, traditional case-based surveillance can present challenges, including incomplete capture of subclinical or unreported cases, making wastewater surveillance (WS) a valuable complementary approach to clinical mpox surveillance (*5*,*6*). Early work demonstrated that viral shedding through urine, feces, respiratory secretions, and skin lesions can result in detectable MPXV in wastewater, supporting wastewater-based epidemiology as a viable approach for monitoring and quantifying community transmission (*2*,*7*,*8*). In 1 study, MPXV detections in wastewater were shown to precede the identification of the clinical cases, highlighting its potential for early outbreak detection (*9*). Another study reported increases in wastewater viral concentrations ahead of reported clinical cases (*5*). Studies have also documented correlations between wastewater MPXV concentrations and incident mpox cases within corresponding sewersheds, supporting the utility of WS as a proxy for community-level transmission (*10*). Finally, wastewater detections in areas without corresponding clinical reports may help identify communities where mpox transmission might otherwise remain undetected because of social stigma, limited clinical recognition, or constrained diagnostic capacity (*5*,*9*).

Recognizing WS as a valuable strategy for early identification of infectious disease activity, the North Carolina Department of Health and Human Services (NCDHHS) integrated mpox monitoring into its statewide WS program. In September 2023, nonvariola orthopoxvirus testing was implemented at selected WS sites across the state. That assay detects orthopoxviruses including vaccinia, cowpox, and all clades of MPXV. Primary sites were facilities in Asheville, Charlotte, Greensboro, Greenville, and Raleigh; additional periodic testing was conducted at selected sites in Cary, Chapel Hill, North Durham, and South Durham. To evaluate mpox WS program in North Carolina, we conducted this study to estimate sensitivity, specificity, positive predictive value (PPV), and negative predictive value (NPV) relative to confirmed clinical cases. 

## Methods

### Study Area and Surveillance Period

We conducted a retrospective diagnostic performance analysis to evaluate WS for detecting MPXV circulation in North Carolina. For the period October 29, 2023–May 31, 2025, we compared wastewater detections from the North Carolina Wastewater Monitoring Network, operated by the NCDHHS, with confirmed clinical mpox cases using a +17-day temporal window. We assumed clinical case reporting to be complete for the purposes of this analysis. The study included data from 16 publicly owned wastewater treatment plants (WWTPs) with defined catchment areas serving a combined population of 2,002,864 persons (Table 1). Facilities were in urban and semiurban regions and consisted of 4 plants in Charlotte, 3 plants each in Cary and Raleigh, and 1 plant each in Asheville, Chapel Hill, Greensboro, Greenville, North Durham, and South Durham. During the study period, 45 confirmed mpox cases occurred within the included sewersheds. We excluded sites sampled through CDC-contracted programs (Verily, https://verily.com) and WastewaterSCAN (https://www.wastewaterscan.org) because of differences in laboratory methods, assays, reporting structures, and sampling frequency.

**Table 1:** Wastewater treatment plant/sewershed characteristics in study of mpox surveillance in wastewater, North Carolina, USA, October 2023–May 2025*

Sewershed	Population served	Days measured	Positive days	Negative days	Clinical cases
Cary 1	84,189	7	2	5	0
Cary 2	74,331	7	0	7	1
Cary 3	75,886	7	1	6	1
Chapel Hill	78,141	2	0	2	0
Charlotte 1	68,685	82	4	78	0
Charlotte 2	182,501	84	6	78	6
Charlotte 3	120,000	78	3	75	11
Charlotte 4	82,158	80	9	71	10
Greensboro	135,821	70	3	67	0
Greenville	89,616	73	2	71	0
MSD Buncombe (Asheville)	173,000	61	2	59	5
North Durham	142,000	7	1	6	0
Raleigh	550,000	83	5	78	11
Raleigh 2	30,655	75	4	71	0
Raleigh 3	7,776	78	3	75	0
South Durham	108,105	7	2	5	0
Total	2,002,864	801	47	754	45

*MSD, Metropolitan Sewerage District.

### Clinical Case Data

We also obtained clinical case data from NCDHHS. A confirmed mpox case was defined according to the Council of State and Territorial Epidemiologists case definition from 2022 (22-ID-10) as detection of nonvariola orthopoxvirus or MPXV DNA in a clinical specimen by PCR in a patient with compatible symptoms (*11*). We defined case date as date of symptom onset. We geocoded cases using residential address and spatially assigned them to sewersheds by using ArcGIS Pro (Esri, https://www.esri.com).

### Wastewater Sample Collection and Laboratory Testing.

Influent wastewater was collected as 24-hour composite samples approximately 1 time/week at each WWTP except for the 3 plants in Cary and those in Chapel Hill, North Durham, and South Durham, which were only sampled selectively over the study period, generally in response to concerns about a confirmed or suspected mpox case in the general area. Samples were transported on ice and processed within 24 hours at the University of North Carolina at Chapel Hill. We quantified MPXV DNA using the CDC Non-variola Orthopoxvirus Generic Real-Time PCR Test (*12*). We originally optimized the assay in a wastewater matrix by conducting a thermal gradient from 62°C–52°C to see which annealing temperature resulted in the greatest separation from positive and negative droplet clusters (Appendix Figure). We reported results as viral gene copies normalized to flow rate and population within the sewershed. We classified samples with concentrations above the assay limit of detection (LOD) as positive (n = 17). We experimentally determined the assay limit of blank (LOB) to be 0 copies/L by using methods adapted from Clinical and Laboratory Standards Institute document EP17 (*13*). Because the LOB was 0, we theoretically estimated the assay LOD to be 500 copies/L, because Poisson distribution requires roughly 3 copies per sample to detect >1 copies with 95% probability (*14*,*15*). Using this strategy, we classified samples with concentrations from one half the LOD to the LOD as trace detections (n = 30).

### Overall Sensitivity, Specificity, PPV, and NPV

We classified wastewater observations as positive or negative on the basis of laboratory results, as described previously. For each wastewater sample, the presence of >1 confirmed clinical case within +17 days in the same sewershed was assessed. We selected a +17-day alignment window to approximate the biologically plausible period during which MPXV might be detectable in wastewater relative to a reported clinical case. Available data suggest that viral shedding is most likely to occur at 4 to 25 days (*16*,*17*) and might begin before diagnosis. Additional delays related to seeking care, laboratory confirmation, case reporting, and wastewater sampling can create temporal offsets between infection and detection. We selected the +17-day window to balance those biological and operational considerations while minimizing misclassification. We defined diagnostic categories as follows: true positive (TP), wastewater-positive and >1 clinical case within +17 days; false positive (FP), wastewater-positive and no clinical case within +17 days; true negative (TN), wastewater-negative and no clinical case within +17 days; and false negative (FN), wastewater-negative and >1 clinical case within +17 days.

We calculated sensitivity (TP/[TP + FN]), specificity (TN/[TN + FP]), PPV (TP/[TP + FP]), and NPV (TN/[TN + FN]) overall and stratified results by WWTP/sewershed catchment. We calculated 95% CIs using Wilson score intervals because several sites had small denominators and proportions near 0 or 1. The primary analysis classified both positive and trace detections as wastewater-positive (n = 47). A secondary sensitivity analysis restricted wastewater-positive observations to samples exceeding the LOD (n = 17). Because clinical case surveillance is an imperfect reference standard and does not capture all infections, TP/FP/TN/FN classifications should be interpreted as concordance with reported cases rather than true infection status. All analyses were conducted in R version 4.2.3 (The R Project for Statistical Computing, https://www.r-project.org).

## Results

During October 29, 2023–May 31, 2025, a total of 801 wastewater samples were collected across 16 WWTPs serving an estimated population of ≈2 million people. Of those, 47 (5.9%) were classified as wastewater-positive (17 above the LOD and 30 trace detections) and 754 (94.1%) were wastewater-negative. During the same period, 45 confirmed mpox cases were reported that fell within sewersheds representing the WWTPs in this study.

Using a +17-day temporal alignment window, we classified 15 wastewater observations as TPs, 32 as FPs, 627 as TNs, and 127 as FNs (Table 2). The overall sensitivity of wastewater detection identifying the presence of >1 temporally aligned reported mpox case within a sewershed was 10.6% (95% CI 6.5%–16.7%). Specificity was 95.1% (95% CI 93.2%–96.5%). The PPV was 31.9% (95% CI 20.4%–46.2%), and the NPV was 83.2% (95% CI 80.3%–85.7%).

**Table 2:** Diagnostic performance metrics of wastewater monitoring in study of mpox surveillance in wastewater, North Carolina, USA, October 2023–May 2025*

Study	TP	FP	TN	FN	Sensitivity, % (95% CI)	Specificity, % (95% CI)	PPV, % (95% CI)	NPV, % (95% CI)
Detections above LOD and trace detections	15	32	627	127	10.6 (6.5–16.7)	95.1 (93.2–96.5)	31.9 (20.4–46.2)	83.2 (80.3–85.7)
Detections above LOD only	2	15	644	140	1.4 (0.4–5.0)	97.7 (96.3–98.6)	11.8 (3.3–34.3)	82.1 (79.3–84.7)

*FN, false negative; FP, false positive; LOD, limit of detection; NPV, negative predictive value; PPV, positive predictive value; TN, true negative; TP, true positive.

Abbreviations:

At the WWTP sewershed level, sensitivity was highest at Charlotte WWTP 2 (25.0%) and ranged from 0% to 25.0% across sites with clinical activity (Table 3). Several sewersheds had no temporally aligned clinical cases during the surveillance period, resulting in undefined sensitivity estimates. Specificity was consistently high across sites, ranging from 71.4% to 100%. PPV varied widely by facility, ranging from <1% to 100%, reflecting differences in case occurrence and sampling frequency. NPV exceeded 69% at all sites with clinical activity and was 100% at several facilities without aligned cases.

**Table 3:** Diagnostic characteristics stratified by wastewater treatment plant/sewershed in study of mpox surveillance in wastewater, North Carolina, USA, October 2023–May 2025

Sewershed	TP	FP	TN	FN	Sensitivity, % (95% CI)	Specificity, % (95% CI)	PPV, % (95% CI)	NPV, % (95% CI)
Cary 1	0	2	5	0	–	71.4 (35.9–91.8)	<1 (0–65.8)	100 (56.6–100)
Cary 2	0	0	7	0	–	100 (64.6–100)	–	100 (64.6–100)
Cary 3	0	1	2	4	<1 (0–49.0)	66.7 (20.8–93.9)	<1 (0–79.3)	33.3 (9.7–70.0)
Chapel Hill	0	0	2	0	–	100 (34.3–100)	–	100 (34.3–100)
Charlotte 1	0	4	78	0	–	95.1 (88.1–98.1)	<1 (0–49.0)	100 (95.3–100)
Charlotte 2	6	0	60	18	25 (12.0–45.0)	100 (93.8–100)	100 (61.0–100)	77.0 (66.4–84.9)
Charlotte 3	3	0	52	23	11.5 (3.5–29.0)	100 (93.1–100)	100 (43.9–100)	69.3 (58.2–78.6)
Charlotte 4	3	6	44	27	10.0 (0.03–25.6)	88.0 (76.2–94.4)	33.3 (12.1–64.6)	62.0 (50.3–72.3)
Greensboro	0	3	67	0	–	95.7 (88.1–98.5)	<1 (0–56.2)	100 (94.6–100)
Greenville	0	2	71	0	–	97.3 (90.6–99.1)	<1 (0–65.8)	100 (94.9–100)
MSD Buncombe (Asheville)	0	2	42	17	<1 (0–18.4)	95.4 (84.9–98.7)	<1 (0–79.3)	71.2 (58.6–81.1)
North Durham	0	1	6	0	–	85.7 (48.7–97.4)	<1 (0–79.3)	100 (61.0–100)
Raleigh	3	2	40	38	7.3 (2.5–19.4)	95.2 (84.2–98.7)	60.0 (23.1–88.2)	51.3 (40.4–62.1)
Raleigh 2	0	4	71	0	–	94.7 (87.1–97.9)	<1 (0–49.0)	100 (94.9–100)
Raleigh 3	0	3	75	0	–	96.2 (89.3–98.7)	<1 (0–56.2)	100 (95.1–100)
South Durham	0	2	5	0	–	71.4 (35.9–91.8)	<1 (0–65.8)	100 (56.6–100)

*Dashes indicate that sensitivity cannot be evaluated in sewersheds with no clinical cases (TP+FN) and PPV cannot be evaluated in sewersheds with no detections (TP+FP). FN, false negative; FP, false positive; MSD, Metropolitan Sewerage District, NPV, negative predictive value; PPV, positive predictive value; TN, true negative; TP, true positive.

When restricting wastewater-positive observations to samples exceeding the LOD (n = 17), sensitivity decreased from 10.6% (95% CI 6.5%–16.7%) to 1.4% (95% CI 0.4%–5.0%), and specificity increased from 95.1% (95% CI 93.2%–96.5%) to 97.7% (95% CI 96.3%–98.6%) (Table 2). The number of FP observations decreased from 32 to 15, whereas FN observations increased from 127 to 140. PPV decreased from 31.9% (95% CI 20.4%–46.2%) to 11.8% (95% CI 3.3%–34.3%), and NPV was similar (83.2% vs. 82.1%). Site-level performance patterns were unchanged.

## Discussion

This evaluation assessed the performance of WS for detecting MPXV across 16 WWTPs in North Carolina during October 2023–May 2025. Using a +17-day alignment window, wastewater detection demonstrated low sensitivity for identifying temporally aligned clinical cases (10.6% overall when including trace detections and 1.4% when restricted to detections above the LOD) but high specificity (>95%) and moderate NPV of ≈83%. We selected a +17-day window to account for the expected duration of MPXV shedding and potential delays between symptom onset, clinical diagnosis, and case reporting, while also accommodating once-weekly wastewater sampling. Because wastewater represents a composite, time-integrated signal and samples are collected weekly, this window reduces misclassification that could arise from temporal offsets between individual infections and wastewater detection.

Those findings compare with national estimates reported in another study (*16*) that observed a daily sensitivity of 13.8% for detecting >1 person shedding MPXV and a weekly sensitivity of 31.7%. The lower sensitivity observed in North Carolina likely reflects differences in analytic approach, case density, and assumptions about viral shedding. Adams et al. (*16*) assumed complete case ascertainment and uniform 25-day viral shedding after symptom onset of mpox, whereas our analysis relied on confirmed case onset dates and a fixed +17-day temporal alignment window. This approach likely provides a more conservative estimate of sensitivity because wastewater detections occurring outside the defined window were classified as discordant even if they reflected prolonged or presymptomatic shedding. In addition, only 45 cases were identified across a population of >2 million persons, limiting the probability that wastewater concentrations would exceed detection thresholds.

Despite low sensitivity for detecting single cases, specificity was high (95%–98%), indicating that wastewater-negative observations were generally concordant with the absence of reported cases. The NPV exceeded 80% overall and was higher at several individual sites, suggesting that the absence of a wastewater detection was associated with a low likelihood of concurrent reported cases. However, the PPV was modest (31.9% in the primary analysis and 11.8% in the LOD-only analysis), reflecting that some wastewater detections occurred without temporally aligned reported cases. Such detections could represent travel-associated cases, delayed reporting, prolonged shedding, asymptomatic or subclinical infections, or infections among persons residing outside the mapped catchment area. As a consequence, wastewater detections occurring without temporally aligned reported cases should not necessarily be interpreted as analytical FPs and might indicate true but unrecognized infections, suggesting that PPV might be underestimated. Therefore, if even a subset of those observations represented true infections, the PPV of WS would be higher than estimated in this analysis.

Of note, sensitivity increased substantially when trace detections were included, indicating that low-level signals contributed meaningfully to temporal concordance with reported cases. That finding underscores the tradeoff between sensitivity and specificity when defining wastewater-positive thresholds. Restricting classification to concentrations above the assay LOD generally increased specificity but markedly reduced sensitivity, potentially limiting the ability to detect low-level community signals in low-incidence settings. In North Carolina, trace detections are interpreted within a structured public health decision framework rather than as definitive evidence of transmission and might prompt notification of local health departments and wastewater partners, review of recent case activity, enhanced syndromic surveillance monitoring, and coordination regarding case investigation or specimen retesting. Those actions are consistent with our public health action decision tree for confirmed detections (Figure). Confirmed detections might also involve escalation actions such as broader public notification, targeted outreach, and intensified sampling.

**Figure:**
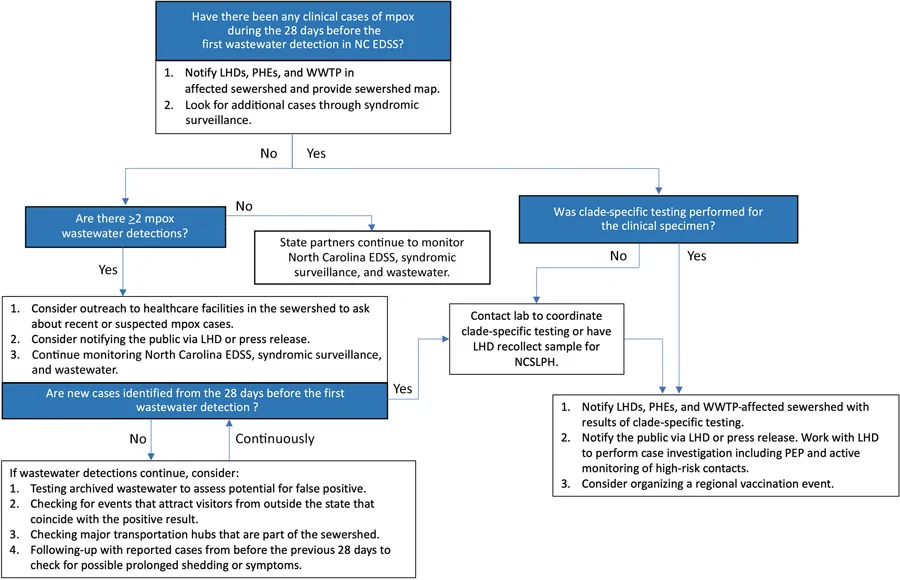
Public Health Action Decision Tree for monkeypox virus surveillance in wastewater, North Carolina, USA, October 2023–May 2025. Period of 28 days is based on typical lesion healing duration (*16*). EDSS, Electronic Disease Surveillance System; LHD, local health department; PEP, postexposure prophylaxis; PHE, public health epidemiologist; SLPH, State Laboratory of Public Health; WWTP, wastewater treatment plant.

Several factors likely contributed to the low sensitivity observed. First, mpox incidence during the study period was low and sporadic, reducing the probability that viral shedding might be detectable within large, pooled wastewater catchments. Second, sampling occurred once per week, which might have missed transient shedding signals. Third, wastewater represents an aggregate population-level signal, and the dilution effect within large catchments (some serving >500,000 persons) might limit detection of individual infections. Fourth, alignment relied on residential address assignment; persons infected but not residing within the sewershed would reduce measured concordance.

The first limitation of this study is that reported clinical cases are not a perfect standard for infection. Our intent was to quantify the extent to which wastewater detections were temporally aligned with reported clinical cases within the same sewershed, rather than to provide an estimate of the true diagnostic accuracy of WS for detecting infection. Second, clinical case reporting was assumed to be complete within each sewershed; however, underascertainment of cases could bias estimates of sensitivity and PPV downward while inflating NPV. Several factors might have contributed to incomplete case detection, including mild or atypical symptoms, barriers to healthcare access, stigma, variation in clinical testing practices, and infections occurring among visitors or commuters whose residence falls outside the wastewater catchment. As a result, wastewater detections might occur in the absence of a temporally aligned reported clinical case.

An additional limitation of this study is that viral shedding dynamics were not directly measured, and the +17-day alignment window might not fully capture the duration or variability of shedding. Wastewater sampling was also conducted only once per week, which might have reduced the likelihood of capturing transient or low-level shedding events and could have contributed to lower observed sensitivity. Finally, although laboratory methods were consistent within this program, sampling frequency and analytic approaches might differ from other surveillance systems and studies, limiting direct comparability with published estimates, including those reported by Adams et al. (*16*).

Although sensitivity for detecting single cases was limited, high specificity and moderate NPV indicate that absence of detection might provide reassurance that widespread community transmission is unlikely. In low-prevalence contexts, isolated wastewater detections should be interpreted cautiously and in conjunction with epidemiologic investigation (*9*). Conversely, sustained nondetection in monitored sewersheds might help inform risk communication and public health response decisions. Continued evaluation across varying transmission intensities and analytic thresholds will help clarify the optimal role of mpox WS in outbreak preparedness and response. Finally, because clinical surveillance does not capture all mpox infections, some wastewater detections classified as FPs might instead represent true but unrecognized infections. Consequently, the sensitivity and positive predictive value reported here should be considered conservative estimates of concordance with reported clinical cases rather than measures of true diagnostic accuracy.

Appendix Additional information about monkeypox virus surveillance in wastewater, North Carolina, USA, October 2023–May 2025.
