# Supplementary material for: Monkeypox Virus Surveillance in Wastewater, North Carolina, USA, October 2023–May 2025
Source: Emerg Infect Dis. 2026 Aug;32(Suppl 1):S93–9. doi: 10.3201/eid3213.260414 (PMC13585670; doi:10.3201/eid3213.260414)
Supplement: Supplementary file 1 [file 26-0414-Techapp-s1.pdf]

Article DOI: https://doi.org/10.3201/eid3213.260414

EID cannot ensure accessibility for supplementary materials supplied by authors. Readers who have difficulty accessing supplementary content should contact the authors for assistance.

Monkeypox Virus Surveillance in Wastewater, North Carolina, USA, October 2023–May 2025

Appendix

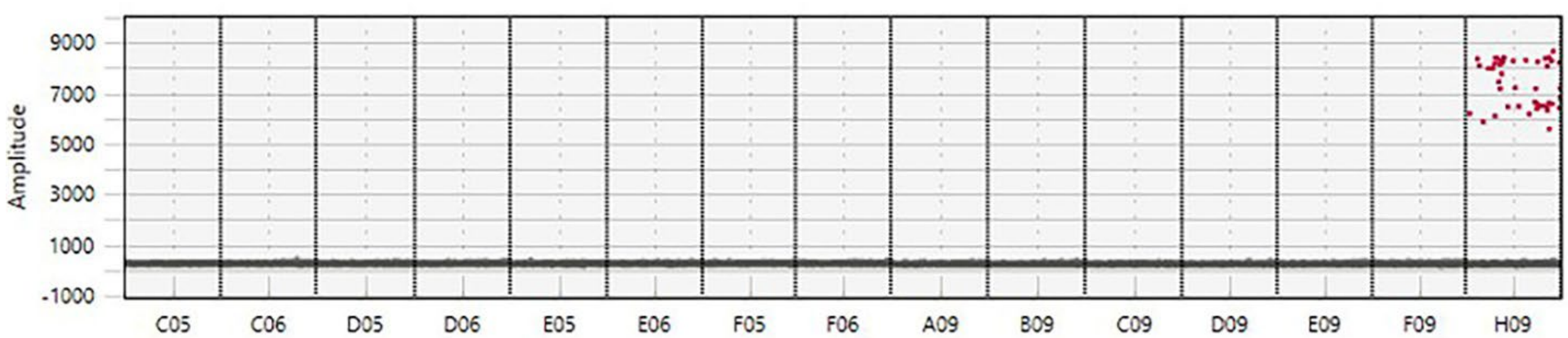

Appendix Figure. NVO method showing the optimized assay in a wastewater matrix. The fluorescence amplitude plot shows the clear separation from negative and positive droplet clusters.
